# Supplementary material for: Inflammaging mediates testosterone declines in men while maintaining high testosterone increases mortality risk
Source: bioRxiv. 2026 Jun 5:2026.06.04.730222. Preprint. [Version 1] doi: 10.64898/2026.06.04.730222 (PMC13252188; doi:10.64898/2026.06.04.730222)
Supplement: 1 [file NIHPP2026.06.04.730222v1-supplement-1.pdf]

bioRxiv preprint doi: https://doi.org/10.64898/2026.06.04.730222; this version posted June 5, 2026. The copyright holder for this preprint (which was not certified by peer review) is the author/funder, who has granted bioRxiv a license to display the preprint in perpetuity. It is made available under a CC-BY 4.0 International license.

Supplementary Table S1. Models predicting cytokines (n = 18,347)

	IL-1β (z)	IL-6 (z)	TNF-α (z)	TNFR1 (z)	GDF-15 (z)
Age (years)	0.001^{t} (0.001)	0.020^{**} (0.001)	0.005^{**} (0.0004)	0.010^{**} (0.0003)	0.032^{**} (0.0005)
High BP or CVD (0,1)	0.032^{**} (0.011)	0.120^{**} (0.014)	0.054^{**} (0.006)	0.068^{**} (0.006)	0.141^{**} (0.008)
Diabetes (0,1)	-0.033^{t} (0.018)	0.093^{**} (0.024)	0.047^{**} (0.011)	0.123^{**} (0.010)	0.615^{**} (0.014)
Cancer (0,1)	-0.037^{t} (0.021)	0.165^{**} (0.028)	0.049^{**} (0.013)	0.091^{**} (0.011)	0.133^{**} (0.016)
BMI (kg/m^2)	0.008^{**} (0.001)	0.051^{**} (0.002)	0.012^{**} (0.001)	0.019^{**} (0.001)	0.015^{**} (0.001)

^{t} $p < 0.10$; * $p < 0.05$; ** $p < 0.01$

bioRxiv preprint doi: https://doi.org/10.64898/2026.06.04.730222; this version posted June 5, 2026. The copyright holder for this preprint (which was not certified by peer review) is the author/funder, who has granted bioRxiv a license to display the preprint in perpetuity. It is made available under a CC-BY 4.0 International license.

Supplementary Table S2. Mediation results for age, diseases, cytokines, and testosterone in men in the UKB (n = 18,347)

Predictor	Mediator	Outcome	Proportion mediated
Age	IL-6	Free T	0.02
High BP or CVD	IL-6	Free T	0.08
Cancer	IL-6	Free T	0.09
BMI	IL-6	Free T	0.11
Age	GDF-15	Free T	0.12
High BP or CVD	GDF-15	Free T	0.37
Diabetes	GDF-15	Free T	0.91
Cancer	GDF-15	Free T	0.18
IL-6	GDF-15	Free T	0.30

bioRxiv preprint doi: https://doi.org/10.64898/2026.06.04.730222; this version posted June 5, 2026. The copyright holder for this preprint (which was not certified by peer review) is the author/funder, who has granted bioRxiv a license to display the preprint in perpetuity. It is made available under a CC-BY 4.0 International license.

Supplementary Table S3. Mediation results for age, diseases, cytokines, and testosterone in men in the UKB (n = 18,347)

Predictor	Mediator	Outcome	Proportion mediated
Age	IL-6	BioA T	0.03
Cancer	IL-6	BioA T	0.13
BMI	IL-6	BioA T	0.13
Age	TNFR1	BioA T	0.02
Diabetes	TNFR1	BioA T	0.13
Cancer	TNFR1	BioA T	0.13
BMI	TNFR1	BioA T	0.06
Age	GDF-15	BioA T	0.11
Diabetes	GDF-15	BioA T	1.00
Cancer	GDF-15	BioA T	0.19
IL-6	GDF-15	BioA T	0.21
TNFR1	GDF-15	BioA T	0.95

bioRxiv preprint doi: https://doi.org/10.64898/2026.06.04.730222; this version posted June 5, 2026. The copyright holder for this preprint (which was not certified by peer review) is the author/funder, who has granted bioRxiv a license to display the preprint in perpetuity. It is made available under a CC-BY 4.0 International license.

Supplementary Table S4. Logistic regression models predicting mortality (OR with 95% CI) (n = 18,347)

	All causes (n = 3,073 deaths)		Cancer (n = 1,090 deaths)		Infection (n = 153 deaths)		Cancer or Infection (n = 1,243 deaths)	
Time (years)	0.76^{**} (0.72, 0.80)	0.76^{**} (0.72, 0.80)	0.86^{**} (0.80, 0.93)	0.86^{**} (0.80, 0.93)	0.94 (0.77, 1.14)	0.94 (0.77, 1.14)	0.87^{**} (0.81, 0.94)	0.87^{**} (0.81, 0.93)
Age (years)	1.09^{**} (1.08, 1.10)	1.09^{**} (1.08, 1.10)	1.07^{**} (1.06, 1.08)	1.07^{**} (1.06, 1.08)	1.07^{**} (1.04, 1.11)	1.07^{**} (1.04, 1.11)	1.07^{**} (1.06, 1.08)	1.07^{**} (1.06, 1.08)
High BP or CVD (0,1)	1.29^{**} (1.18, 1.41)	1.29^{**} (1.18, 1.42)	1.03 (0.90, 1.17)	1.03 (0.90, 1.18)	1.46^{*} (1.03, 2.10)	1.46^{*} (1.03, 2.10)	1.07 (0.94, 1.22)	1.07 (0.94, 1.22)
Diabetes (0,1)	1.06 (0.91, 1.23)	1.06 (0.91, 1.23)	0.80^{*} (0.63, 1.00)	0.80^{t} (0.63, 1.00)	1.28 (0.80, 2.01)	1.27 (0.79, 2.00)	0.87 (0.71, 1.07)	0.87 (0.71, 1.07)
Cancer (0,1)	1.67^{**} (1.43, 1.96)	1.67^{**} (1.43, 1.96)	2.50^{**} (2.07, 3.01)	2.50^{**} (2.07, 3.01)	1.08 (0.59, 1.84)	1.08 (0.59, 1.84)	2.35^{**} (1.95, 2.81)	2.35^{**} (1.95, 2.81)
BMI (kg/m^2)	0.99^{*} (0.98, 1.00)	0.99^{*} (0.98, 1.00)	0.99 (0.97, 1.01)	0.99 (0.97, 1.01)	1.02 (0.99, 1.06)	1.02 (0.99, 1.06)	0.99 (0.98, 1.01)	0.99 (0.98, 1.01)
IL-1β (z)	1.09^{**} (1.02, 1.17)	1.09^{*} (1.02, 1.17)	1.12^{*} (1.02, 1.24)	1.12^{*} (1.02, 1.24)	0.84 (0.64, 1.09)	0.84 (0.64, 1.09)	1.08^{t} (0.99, 1.19)	1.08^{t} (0.99, 1.19)
IL-6 (z)	1.23^{**} (1.17, 1.29)	1.22^{**} (1.16, 1.29)	1.16^{**} (1.08, 1.24)	1.16^{**} (1.08, 1.24)	1.27^{**} (1.08, 1.46)	1.27^{**} (1.08, 1.46)	1.18^{**} (1.11, 1.26)	1.18^{**} (1.11, 1.26)
TNF-α (z)	1.01 (0.89, 1.14)	1.01 (0.90, 1.15)	0.94 (0.78, 1.12)	0.94 (0.78, 1.12)	0.92 (0.57, 1.40)	0.92 (0.57, 1.40)	0.93 (0.78, 1.10)	0.93 (0.78, 1.10)
TNFR1 (z)	1.06 (0.92, 1.23)	1.06 (0.91, 1.23)	0.91 (0.74, 1.12)	0.91 (0.74, 1.12)	1.22 (0.76, 1.94)	1.22 (0.76, 1.94)	0.96 (0.79, 1.16)	0.95 (0.78, 1.16)
GDF-15 (z)	2.96^{**} (2.69, 3.27)	2.96^{**} (2.69, 3.26)	1.89^{**} (1.66, 2.14)	1.89^{**} (1.67, 2.15)	1.85^{**} (1.38, 2.43)	1.84^{**} (1.38, 2.42)	1.94^{**} (1.72, 2.18)	1.94^{**} (1.72, 2.19)
Low Free T	1.09 (0.98, 1.20)		1.10 (0.95, 1.28)		0.95 (0.65, 1.41)		1.08 (0.94, 1.25)	
High Free T	1.12^{t} (0.99, 1.25)		1.12 (0.95, 1.33)		1.26 (0.81, 1.94)		1.14 (0.97, 1.34)	
Low BioA T		1.14^{*} (1.02, 1.26)		1.05 (0.91, 1.23)		1.01 (0.69, 1.49)		1.05 (0.91, 1.21)
High BioA T		1.17^{**} (1.04, 1.32)		1.12 (0.95, 1.33)		1.27 (0.81, 1.98)		1.15^{t} (0.98, 1.35)

^{t} $p < 0.10$; * $p < 0.05$; ** $p < 0.01$

bioRxiv preprint doi: https://doi.org/10.64898/2026.06.04.730222; this version posted June 5, 2026. The copyright holder for this preprint (which was not certified by peer review) is the author/funder, who has granted bioRxiv a license to display the preprint in perpetuity. It is made available under a CC-BY 4.0 International license.

Supplementary Table S5. Logistic regression models predicting mortality (OR with 95% CI) (n = 18,347)

	Cardiovascular (n = 763 deaths)		Prostate cancer (n = 126 deaths)		Cancer, excluding prostate (n = 964 deaths)	
Time (years)	0.75** (0.68, 0.82)	0.75** (0.68, 0.82)	0.93 (0.75, 1.16)	0.93 (0.75, 1.16)	0.86** (0.79, 0.93)	0.86** (0.79, 0.93)
Age (years)	1.07** (1.05, 1.08)	1.07** (1.05, 1.08)	1.10** (1.06, 1.14)	1.10** (1.07, 1.14)	1.07** (1.06, 1.08)	1.07** (1.06, 1.08)
High BP or CVD (0,1)	1.86** (1.57, 2.20)	1.86** (1.57, 2.20)	0.89 (0.61, 1.30)	0.90 (0.61, 1.30)	1.05 (0.91, 1.21)	1.05 (0.91, 1.21)
Diabetes (0,1)	1.39** (1.11, 1.72)	1.39** (1.11, 1.72)	0.60 (0.29, 1.13)	0.60 (0.29, 1.14)	0.83 (0.65, 1.05)	0.83 (0.65, 1.05)
Cancer (0,1)	0.77 (0.56, 1.04)	0.77t (0.56, 1.04)	3.16** (1.97, 4.89)	3.16** (1.97, 4.89)	2.31** (1.88, 2.81)	2.31** (1.88, 2.81)
BMI (kg/m^2)	1.01 (1.00, 1.03)	1.01 (1.00, 1.03)	1.04t (0.99, 1.09)	1.04t (1.00, 1.09)	0.98t (0.97, 1.00)	0.98t (0.97, 1.00)
IL-1β (z)	1.04 (0.92, 1.16)	1.04 (0.92, 1.16)	1.00 (0.74, 1.32)	1.00 (0.74, 1.32)	1.14* (1.03, 1.26)	1.14* (1.03, 1.26)
IL-6 (z)	1.12** (1.03, 1.22)	1.12** (1.03, 1.22)	0.89 (0.69, 1.11)	0.89 (0.70, 1.11)	1.19** (1.11, 1.27)	1.19** (1.11, 1.27)
TNF-α (z)	0.98 (0.79, 1.20)	0.98 (0.79, 1.20)	0.83 (0.47, 1.37)	0.83 (0.47, 1.37)	0.96 (0.79, 1.15)	0.96 (0.79, 1.15)
TNFR1 (z)	1.29* (1.03, 1.62)	1.29* (1.03, 1.62)	0.56t (0.30, 1.02)	0.55t (0.30, 1.02)	0.98 (0.78, 1.21)	0.97 (0.78, 1.21)
GDF-15 (z)	2.03** (1.76, 2.34)	2.01** (1.75, 2.32)	2.05** (1.45, 2.82)	2.07** (1.46, 2.84)	1.82** (1.59, 2.07)	1.82** (1.60, 2.08)
Low Free T	0.90 (0.75, 1.07)		1.20 (0.79, 1.85)		1.08 (0.92, 1.27)	
High Free T	0.91 (0.74, 1.12)		1.21 (0.74, 1.96)		1.10 (0.92, 1.32)	
Low BioA T		1.10 (0.92, 1.31)		1.01 (0.67, 1.54)		1.06 (0.90, 1.24)
High BioA T		1.07 (0.86, 1.32)		1.11 (0.69, 1.79)		1.12 (0.94, 1.34)

t $p < 0.10$; * $p < 0.05$; ** $p < 0.01$
